# Assessing Student Perceptions of a Norwegian University's COVID-19 Response Strategy: A Cross-Sectional Study

**DOI:** 10.3389/fpubh.2021.700542

**Published:** 2021-08-20

**Authors:** Fiona Vande Velde, Ahmed Hamed, Joakim Slinning Lange, Turid Sælid, Sheri Bastien

**Affiliations:** ^1^Department of Public Health Science, Faculty of Landscape and Society, Norwegian University of Life Sciences, Ås, Norway; ^2^Department of Community Health Sciences, Cumming School of Medicine, University of Calgary, Calgary, AB, Canada

**Keywords:** pandemic, higher education institutions, health promotion, public health emergency response, nudging, risk perception, trust

## Abstract

**Aim:** This study aims to investigate Norwegian students' perceptions toward a higher education institution (HEI)'s COVID-19 response strategy, differentiating between three behavioral techniques: informing (i. e., email updates about COVID-19), nudging (i.e., visual cues as reminders), and creating novel opportunities (i.e., provision of antibacterial dispensers). In addition, the study assesses to what extent these perceptions are influenced by COVID-19 related psychological factors: risk perception; attitudes toward infection prevention and control (IPC) behaviors; perceived behavior control; institutional trust.

**Methods:** A cross-sectional online survey was conducted among a student population. The survey was developed to evaluate the HEI's response strategy, and distinct perceptions of COVID-19 and related practices. Structural equation modeling (SEM) was applied to estimate the effect of the psychological factors on the attitude toward different behavioral techniques.

**Results:** Creating novel opportunities was perceived most positively from the students, secondly, informing the students through email updates about COVID-19, finally, reminders through visual cues. Institutional trust presented the largest positive effect on informing the students through email updates, while no effect was measured for reminders. Attitudes toward IPC behaviors showed the strongest effect on students' perceptions of new opportunities and reminders, whereas providing email updates about COVID-19 is less affected by pre-existing perceptions.

**Conclusions:** A host of factors such as institutional trust, and perceptions concerning IPC measures and risk severity, influence students' perceptions of different behavior change techniques. This type of knowledge can contribute to understanding how perceptions can impact acceptance and adoption of specific preventive measures within a pandemic response. An assessment as such may result in more ethical and relevant future efforts.

## Introduction

The COVID-19 pandemic has had a significant impact on the health, well-being and behaviors of students, and the general population, globally (1). As the pandemic escalated, it led to the total or partial closure of many higher education institutions (HEIs) campuses, and following, a complete reorganization of their activities including reorienting classes to a digital format. In Norway, all HEIs suspended their on-campus activities on March 12th 2020 (2), until the end of that semester in June. To reopen campus for students next school year in August, HEIs implemented a mitigation strategy recommended by the Norwegian Institute of Public Health (Folkehelseinstituttet, FHI) (3). The response strategy relied exclusively on non-pharmaceutical interventions, thus, staff and students' compliance with infection prevention measures, mainly focussing on good hygiene (e.g., handwashing, adopting a coughing-etiquette), physical distancing (e.g., keeping 1 meter distance, staying home when sick), and frequent cleaning of high-contact surfaces (3). In Norwegian context, wearing face masks was at that moment not included in the recommendations.

Effective and ethical public health emergency responses are informed by behavioral science, therefore, response strategies should by extension be theoretically and empirically informed (4). Since the outset of the pandemic, a growing number of studies have focussed on knowledge, attitude and practices measurements of COVID-19 infection prevention and control (IPC) behaviors among student populations globally (5). In general, findings from these studies indicate students' positive knowledge, attitudes and practices of IPC behaviors to mitigate the spread of COVID-19. However, there are limitations of relying solely on knowledge and cognitive attitudes that are anchored in assumptions of rationality for understanding actual behavior. In particular, repetitive behaviors such as handwashing, have been proposed to function through low-processing mechanisms such as heuristics (i.e., mental shortcuts) and automatic processes (i.e., unconscious habits) (6). One study with a student population in the UK found that the strongest predictor for handwashing behavior during this pandemic was self-reported habit (7). Therefore, HEIs should include a combination of behavioral techniques in their response strategy, targeting all routes to increase students' compliance with IPC measures. Different techniques include, but are not restricted to, informing, nudging [i.e., altering the environment in a meaningful way to shape peoples' behavior, without depriving them of choice or providing economic incentives (8)], or providing a novel opportunity (e.g., placing an antibacterial dispenser in a strategic location). Based on Hansen's definition of a nudge [(9), p. 174], the latter should be regarded as two distinct techniques: “…Thus a nudge amongst other things works independently of: (i) forbidding or adding any rationally relevant choice options, (ii) changing incentives, in terms of time, effort required, social sanctions, economic and so forth, or (iii) the provision of factual information and rational argumentation.” Adding a rational choice option is considered as a novel opportunity in which people can engage in a certain behavior, which they could not have engaged in before. These three techniques were, amongst others, implemented by the HEI in question for our student population, and therefore included in this study: email updates about COVID-19; reminders to perform IPC measures as nudges through posters, stickers and screensavers; and provision of antibacterial dispensers near building entrances and in classrooms.

In a review of studies of attitudinal determinants of protective behaviors during the 2009 influenza pandemic, satisfaction with the communications received about the disease by the target population was associated with compliance with preventive, avoidant, and management behaviors (10). This highlights the importance of evaluating perceptions of the implemented response strategies. Moreover, such an assessment may result in more ethical and relevant future efforts (11). To date, most research has focussed on understanding human behavior for tailoring response strategies, but to our knowledge, fewer studies have attempted to evaluate the perceptions toward these strategies. This is especially relevant given that certain strategies are set up to encourage students to comply with IPC behaviors in a less conscious mode (e.g., nudging). Therefore, ethical concerns may arise from applying these strategies, without consent or support from the receiving population. Engelen proposed a framework for assessing ethical aspects of nudges in health promotion (12). The main categories of the assessment can be applied more broadly to other types of behavioral techniques and include the evaluation of various aspects of an intervention. The framework identifies three main categories for evaluating an intervention: ends (i.e., evaluation of an individual's goals and values), means (i.e., evaluation of an individual's decision-making process) and agents (i.e., evaluation of an individual's trust toward the implementers) (11). The three categories can be interlinked with COVID-19 related psychological factors for IPC behaviors: attitudes toward IPC behaviors (i.e., ends); risk perception and perceived behavior control (i.e., means); institutional trust (i.e., agents). Engelen's framework was used to conceptualize these psychological factors, and underpin the evaluation of a COVID-19 response strategy. To our knowledge, established perceptions of COVID-19 and related IPC behaviors have not been explored in the context of attitudes toward behavioral techniques. We believe that their perceptions of COVID-19 will influence their evaluation of received responses. Therefore, we anticipate these factors will influence students' perceptions on the different behavioral techniques implemented by a HEI, and seek to establish an explanatory model through this study.

Attitudes toward IPC behaviors. An intervention is considered more legitimate and democratic, hence receiving more support, if the targeted behavior generates more health benefits and is underpinned by population preferences (12). A review of studies found positive attitudes toward the proposed IPC behaviors (e.g., washing hands, social distancing) recommended to mitigate the spread of COVID-19 by students, and the population in general (5).

Risk perception. Threat appraisal and risk perception are known to be important determinants of the public's willingness to cooperate and adopt IPC behaviors during pandemics, including frequent hand washing, physical distancing, avoiding public places, and wearing face masks (10, 13). The current pandemic has also resulted in a vast amount of research aimed at gauging the effect of risk perception on the adoption of recommended practices, and findings vary across settings and populations (14).

Perceived behavioral control. Some behavioral techniques (e.g., nudging) have been criticized previously, on the basis that such approaches are paternalistic and limit an individual's autonomy and decision to engage in a behaviour (15). However, it is also argued that this can be mitigated if the intervention or proposed behavior change strategy is implemented in a transparent, easy to resist manner, which may to some extent preserve an individual's autonomy and therefore more supported (12, 16).

Institutional trust. Pervious pandemics have shown a positive effect of public and governmental trust on people's willingness to adopt recommended behaviour (10). However, current research on the COVID-19 pandemic has presented mixed findings concerning the effect of trust on compliance with recommended and voluntary practices (17, 18). Nevertheless, trust plays an important role when disseminating information or implementing certain behavioral techniques (12, 19).

In summary, this study aims to investigate Norwegian students' attitudes toward a HEI COVID-19 pandemic response strategy, differentiating between three different behavioral techniques: informing, nudging, and creating novel opportunities. In addition, the study will assess to what extent these attitudes are influenced by COVID-19 related psychological factors: attitudes toward IPC behaviors (i.e., ends); perceived behavior control and risk perception (i.e., mean); institutional trust (i.e., agents).

## Materials and Methods

### Study Design, Population, and Data Collection

A cross-sectional online survey was conducted in the context of a course focused on participatory approaches in public health, emphasizing the importance of including a stakeholders' perspective when implementing a response strategy. The study aims to give a broad overview of, and map salient issues with perceptions of an institutional Covid-19 response strategy. A survey is an appropriate method for investigating perceptions among a large cross-section of the student body. The entire student population at one HEI in Norway (*n* = 5,158) was considered for inclusion, since this particular sample was exposed to the HEI's COVID-19 response strategy. Students were recruited through the HEI's email updates on the COVID-19 situation. The emails contained an invitation and link to the online survey, from which they could complete the survey either in Norwegian or English. The request to participate in the study was sent out twice, first in October and then in November 2020. To increase the response rate, and at the same time reduce response bias toward students that are more concerned about COVID-19, an incentive was provided that consisted of a lottery for one book voucher (NOK 350) and 5 coffee-vouchers (value of 5 cups) from the local café, which was open at that moment. Participants were eligible if they were exposed to the HEI on-campus interventions during the period it was open from August-October 2020, which was probed at the beginning of the survey.

### Survey Design

The survey was developed to evaluate the HEI's response strategy based on Engelen's framework (12), and measured four distinct perceptions of COVID-19 and related practices. The framework and defined variables guided the purpose of the study and design of the instrument. Firstly, COVID-19 risk and the perception of IPCs: risk severity (4 items) and risk susceptibility (2 items) (20), and attitudes toward IPC behaviors (12 items) (21). Secondly, perceptions toward the HEI: institutional trust (4 items) (19). Thirdly, perceptions toward the HEI's response strategy: attitudes toward reminders (6 items), attitudes toward novel opportunities (4 items), attitude toward email updates about COVID-19 (2 items) (21) and perceived behavioral control (12 items) (21). Finally, an open field was provided to encourage students to express concerns or suggestions related to the HEIs response strategy. However, much of the students' responses related to other impacts COVID-19 had on their study ability. Therefore, results from this section are omitted from the study's analysis, for purposes of keeping a focussed paper.

The items and corresponding constructs of the factors are presented in Supplementary file 1. The items were measured on a 6-point bipolar scale (e.g., necessary–unnecessary), or on a 6-point Likert scale in which the respondents were requested to indicate their perception to a statement on a scale ranging from strongly disagree to strongly agree. The utility in six responses rather than five, or more generally, an even number of options rather than an odd number, is the elimination of a middle choice that often gives respondents an unintentional respite that provides researchers with little useful data. Moreover, a recent study measuring psychometric perspectives provided more accurate statistical results when implementing a 6-point scale (22). The survey did not include any demographic questions in order to ensure full anonymity. According to Norwegian law, data that is fully anonymized is not required to obtain approval from the Norwegian center for research data (NSD)^1^, as well as exempted from ethical obligations toward the Regional Committees for Medical and Health Research Ethics (REK)^2^ Nevertheless, we received support to implement the study by the University leadership and the COVID-19 response team. The survey was developed in both Norwegian and English, and back-translated for consistency. A pilot survey was pre-tested by 3 PhD-students and one Postdoctoral fellow at the Department of Public Health Science at the HEI, and questions were adapted to increase the comprehension. The final version of the survey was administered through an anonymous online system (nettskjema.no, 2020, Nettskjema UiO).

### Statistical Analyses

Responses were coded in a database using the Statistical Package for the Social Sciences (SPSS, IBM SPSS Statistics version 25.0). Firstly, we assessed the respondents' exposure to on-campus interventions, which were subsequently omitted from further analysis if they provided a negative response. Secondly, to pool the data from both the Norwegian and the English survey, we performed a Levene' s test to assess the equality of variance, based on the median for robustness.

Finally, structural equation modeling (SEM) was applied to estimate the effect of the psychological factors of the different behavioral techniques. SEM was performed using the lavaan package (23) in the statistical software R (lavaan version 0.6-7, R version 3.5.2, The R Foundation for Statistical Computing, 2016). The maximum likelihood estimation was used to assess for missing values, using the Yuan-Bentler correction. First, we inspected the baseline model through a confirmatory factor analysis (CFA), without specification of interactions between factors (i.e., latent variables). CFA allowed us to detect irregularities in the observed data such as unsuitable factor loadings (< 0.60) and insignificant variances, and exclude if necessary. Afterwards, SEM was evaluated using the proposed interactions between the included factors, and model fit acquired using following indices: the Comparative Fit Index (CFI) and the Tucker Lewis Index (TLI) (CFI/TLI > 0.90), the Root Mean Square of Approximation (RMSEA) (< 0.08) and the Standard Root Mean Square Residual(SRMR) (< 0.10) (24).

## Results

### Summary of the Responses

A total of 5,158 students receive the email updates about COVID-19 by the HEI, and accordingly the invitation to participate in the study. We registered 359 completed surveys, thus a 7% response rate, from which 327 students filled in the Norwegian version and 32 students the English version. Thirteen students responded they were not exposed to any on-campus activities and were excluded from further analysis. This resulted in 317 Norwegian- and 29 English- surveys (Supplementary file 2), which were compared for variance equality. Levene's test showed inequal variance for one item corresponding to the factor risk severity [Q3_5, *F*_(1, 338)_ 10.90, *p* = 0.001]. Supplementary file 3 presents the Levene's test for all items included in the survey. We excluded Q3_5 and pooled both datasets for further analyses. The scale of the dataset allows us to perform the proposed analysis, however, the response rate limits us to interpret the results for the whole student population. We therefore position the results as being informative rather than representable for our population of interest.

### Modeling the Data

We inspected the baseline model through CFA in 2 rounds and the results are presented in Table 1, showing the included factor loadings. Half of the items corresponding to the factor perceived behavioral control had to be excluded from the model due to unsuitable factor loadings below 0.60 (Q10_1, Q10_3, Q10_5, Q10_7, Q10_9, Q10_11). In addition, other items corresponding to attitudes toward IPC behaviors (Q4_1, Q4_2, Q4_4, Q5_4, Q4_6), attitudes toward reminders (Q8_6), and attitudes toward novel opportunities (Q8_5, Q9_5) presented factor loadings below 0.60 and were excluded from further analyses. The factor risk susceptibility had to be fully removed from the model due to the corresponding items' insignificant variance (Q3_3, Est = 0.48, se = 0.29 *p* = 0.104, Q3_6, Est = 0.15, se = 0.44 *p* = 0.725).

**Table 1:** Confirmatory factor analysis.

**Factor** ** label**	**Item** ** label**	**Factor** ** estimate**	**Std. Err**	**Std.** ** loading**
Risk severity	Q3_1	1.000		0.732
	Q3_2	1.331	0.198	0.653
	Q3_4	1.262	0.151	0.712
Risk susceptibility	Q3_3	1.000		0.795
	Q3_6	1.137	0.373	0.896
Attitudes IPC behaviours	Q4_1	1.000		0.397^a^
	Q5_1	1.212	0.196	0.659
	Q4_2	1.059	0.153	0.620^b^
	Q5_2	0.976	0.190	0.741
	Q4_3	1.610	0.264	0.673
	Q5_3	1.591	0.310	0.732
	Q4_4	0.503	0.155	0.568^a^
	Q5_4	0.516	0.167	0.572^a^
	Q4_5	1.690	0.405	0.638
	Q5_5	1.648	0.379	0.698
	Q4_6	1.327	0.189	0.527^a^
	Q5_6	1.241	0.199	0.676
Institutional trust	Q7_1	1.000		0.871
	Q7_2	0.903	0.040	0.891
	Q7_3	0.693	0.095	0.806
	Q7_4	0.803	0.089	0.799
Attitudes reminders	Q8_1	1.000		0.687
	Q9_1	1.229	0.088	0.778
	Q8_2	1.117	0.093	0.779
	Q9_2	1.224	0.132	0.763
	Q8_6	0.769	0.091	0.546^a^
	Q9_6	1.079	0.133	0.669
Attitudes opportunities	Q8_3	1.000		0.798
	Q9_3	1.137	0.093	0.855
	Q8_5	1.180	0.246	0.543^a^
	Q9_5	1.329	0.233	0.655^b^
Attitudes emails	Q8_4	1.000		0.787
	Q9_4	0.787	0.106	0.684
Perceived behavioral control	Q10_2	1.000		0.656
	Q10_4	1.138	0.146	0.727
	Q10_6	1.211	0.133	0.717
	Q10_8	1.728	0.235	0.795
	Q10_10	1.576	0.196	0.811
	Q10_12	1.764	0.237	0.862
	Q10_1	0.121	0.119	0.077^a^
	Q10_3	0.055	0.129	0.033^a^
	Q10_5	0.049	0.038	0.063^a^
	Q10_7	0.063	0.106	0.040^a^
	Q10_9	0.148	0.073	0.135^a^
	Q10_11	0.189	0.148	0.098^a^

*Factor loadings of the items included in the baseline model.*

a *items excluded from further analysis due to low factor loadings round 1.*

b *items excluded from further analysis due to low factor loadings round 2*.

**Figure 1:**
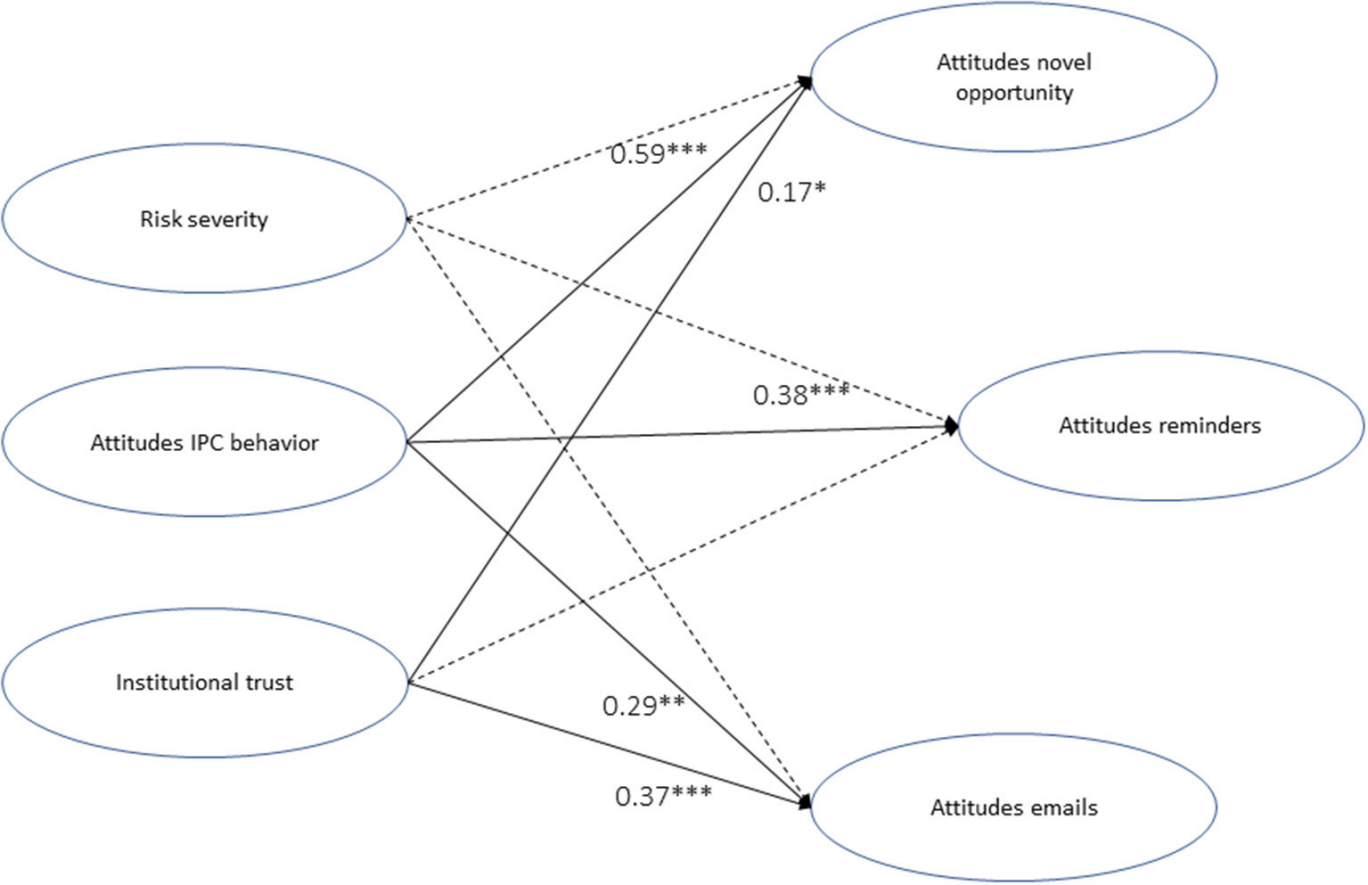
The structural equation model for explaining student perceptions of a Norwegian university's COVID-19 response strategy. Notes, ***p < 0.001, **p < 0.01, *p < 0.05. Figure presents standardized estimates. Full lines present significant correlations, dotted lines present non-significant correlations.

With the remaining items we developed the SEM, which resulted in an acceptable model fit: CFI = 0.88, TLI = 0.86., RMSEA = 0.07, SRMR = 0.05. CFI/TLI are slightly below the proposed fit indices (> 0.90), which is due to an unstable factor of attitudes toward IPC behaviors. However, we did not seek to re-specify the factor, since we aimed at obtaining a general perception toward this set of actions rather than a statistically powerful construct. Re-specifying the factor would result in a loss of information. The final model explained 0.79 of the variance in attitudes toward reminders (AR), 0.62 of the variance in attitudes toward novel opportunities (AO), and 0.74 of the variance in attitudes toward email updates about COVID-19 (AE), and is presented in Figure 1.

### Students' Perceptions

The model presented a significant positive effect of attitudes toward IPC behaviors for the response strategy in general. The effect was largest for AO (β = 0.59, *z* = 5.38, *p* < 0.001), secondly for AR (β = 0.38, *z* = 3.51, *p* < 0.001), and lowest for AE (β = 0.29, *z* = 2.93, *p* = 0.003). Institutional trust had a strong positive effect on AE (β = 0.37, *z* = 3.95, *p* < 0.001), and a moderate effect on AO (β = 0.17, *z* = 2.36, *p* = 0.018), but no significant effect on AR (β = 0.11, *z* = 1.72, *p* = 0.086). In addition, the model showed no significant correlations of perceived behavioral control on the attitudes toward the pandemic response strategy in general (AR, β = −0.01, *z* = −0.1, *p* = 0.92; AO, β = 0.03, *z* = 0.52, *p* = 0.61, AE, β = 0.02, *z* = 0.27, *p* = 0.79). Similarly, risk severity presented no significant effect on the attitudes toward the response strategy in general (AR, β = 0.12, *z* = 1.50, *p* = 0.13; AO, β = 0.00, *z* = 0.02, *p* = 0.98, AE, β = 0.14, *z* = 1.53, *p* = 0.13). However, risk severity presented a strong covariance with attitudes toward IPC behaviors (β = 0.48, *z* = 4.13, *p* < 0.001), and institutional trust (β = −0.22, *z* = −3.26, *p* < 0.001), suggesting an indirect effect on the dependent variables (AR, AO, AE).

To obtain a sense of the magnitude of the dependent variables, Table 2 presents the items' intercepts on a 6-point scale. The intercepts ranging from 1 till 3 can be perceived as negative, and those ranging from 4 to 6 as positive. Results suggest a highest positive attitude for AO, secondly for AE, and the lowest for AR.

**Table 2:** Item intercepts of the dependent variables included in the structural equation model.

**Factor label**	**Item label**	**Intercept estimate**	**Std. Err**
Attitudes reminders	Q8_1	4.478	0.075
	Q9_1	4.398	0.082
	Q8_2	4.373	0.074
	Q9_2	4.338	0.083
	Q9_6	4.207	0.084
Attitudes opportunities	Q8_3	5.704	0.034
	Q9_3	5.732	0.037
Attitudes emails	Q8_4	5.159	0.060
	Q9_4	5.325	0.057

## Discussion

This study measured Norwegian students' perceptions toward the COVID-19 pandemic response strategy implemented by their HEI. Results suggested that creating novel opportunities such provision of antibacterial dispensers in a convenient place was well-received by the students. This intervention could respond to both the novelty and surprise of the action (i.e., creating a new action in an unexpected environment), as well as its convenience. Theories of motivation, particularly of intrinsic motivations and attitudes, place novelty and surprise among the primary factors that arouse interest and motivate exploratory or avoidance behaviour (25). However, both effects could attenuate rather quickly, and subsequently the motivation to engage in a behavior. Therefore, it is important to consider the time-constrained benefits when implementing similar interventions.

Trust toward the institution presented more favorable attitudes in relation to the email updates about COVID-19 (i.e., high-processing interventions), thus information is perceived more positively when provided from a trustworthy source. To a lesser extent, creating novel opportunities, in our case chemical substances such as antibacterial gel, should also be implemented by trustworthy source in order to be perceived as acceptable. This is somewhat in line with a previous study amongst youth in Norway, being informed and trusting the information was deemed important and decreased their anxiety (26). Finally, reminders were not affected by trust toward the source since attitudes are supposedly established for known information, regardless of who provides it. These results present the importance of trust in a source when providing information, however, this trust becomes less relevant for lower-processing interventions such as novel opportunities and reminders.

On the other hand, these low-processing interventions benefit more from pre-existing positive perceptions toward the behaviors they encourage. Attitudes toward IPC behaviors showed the strongest effect on students' perceptions of new opportunities and reminders. Whereas, providing email updates about COVID-19 is less affected by these pre-existing perceptions. This finding indicates the openness of students when receiving new information, not being directed by their already formed opinions.

Risk severity showed no immediate effect on the perception of the overall pandemic response strategy, somewhat in line with a previous study in Norway where only limited predictions of perceived individual risk on the proposed health protective behaviors was found (27). However, an indirect effect was indicated through trust toward the institution and attitudes toward IPC behaviors. Risk severity had a negative effect on trust toward the institution, subsequently resulting in less positive attitudes toward the informative emails and the creation of novel opportunities. On the other hand, risk severity positively influenced attitudes toward IPC behaviors, which accordingly benefitted low-processing interventions. To summarize, students that perceived COVID-19 as a high risk were less receptive toward email updates about COVID-19, while being more receptive toward the reminders. The correlation amongst risk perception, trust and information has been reported consistently during this pandemic (18, 28). However, none of these studies reported the effect of risk perception on different behavioral techniques. Although our results are merely suggestive, they provide a compelling case for further, more rigorous investigation of these associations.

Several limitations of the study must be acknowledged since these have implications on the interpretation of the results. First, our response rate is rather low and we are therefore unable to generalize the results for the whole student population. Although we provided a modest incentive, providing a larger incentive could have resulted in participant bias. Therefore, our results should be regarded as informative rather than representative of a whole population. We encourage replication and further qualitative and quantitative research on this topic. Furthermore, due to the time-sensitive period we were unable to pilot the survey quantitively, including a large enough sample to identify potential issues within constructs. Although we developed the survey on the basis of validated constructs, some scales would have benefited from more rigorous pre-testing (e.g., risk susceptibility). We believe this information could have given a more nuanced view of certain factors as well as provide additional information. Additionally, we analyzed the open-ended question to determine if it would add more depth to our quantitative results, however, much of the responses related to virtual teaching and examination anxiety due to the pandemic situation. Although touching on important aspects, these responses do not add information to the phenomena of interest within this study. Nevertheless, these answers point toward the true concerns of our sample, and it could be relevant to broaden the scope of this line of work by including an analysis of the impact of COVID-19 on different but related issues, such as students' digital literacy in higher-educational (29). Adapting education and communication strategies by HEI's will have an impact on students' preferences and acceptance of a pandemic response strategy (30), therefore, it would be beneficial to include these perspectives in future research.

The findings of this study are important from both an academic and policy perspective. The findings highlight the importance of understanding the perceptions among the target population, in this case students, of a pandemic response strategy implemented by a HEI. Providing new opportunities to engage in recommended preventive measures are highly encouraged, however these should be regularly altered to ensure their durability. Furthermore, a host of factors such as institutional trust, perceptions concerning IPC measures and risk severity influences students' perceptions of different behavior change techniques, and should therefore be considered when developing pandemic response strategies, as well as public health and health promotion strategies more generally. Finally, an emerging body of COVID-19 research has explored and explained behaviors during a pandemic. However, there is a paucity of research thus far that has focussed on the target population's perceptions of an institutional COVID-19 pandemic response strategy. This type of knowledge can contribute to understanding how perceptions can impact acceptance and adoption of specific IPC measures within a pandemic response, and illustrates the importance of pre-testing messages and conducting formative research to ensure appropriate message framing and relevance to the target population. Longer-term studies investigating the effectiveness of specific preventive measures and attenuation of effects over time, as well as ongoing studies of target population needs, preferences, perceptions and uptake of recommended measures are urgently needed to inform policy and practice and ensure the effectiveness and sustainability of COVID-19 response strategies.

## Data Availability Statement

The original contributions presented in the study are included in the article/Supplementary Material, further inquiries can be directed to the corresponding author/s.

## Ethics Statement

Ethical review and approval was not required for the study on human participants in accordance with the local legislation and institutional requirements. Written informed consent for participation was not required for this study in accordance with the national legislation and the institutional requirements.

## Author Contributions

FVV and SB contributed to the data analysis and interpretation, and wrote the manuscript. All authors contributed equally to the research design and data collection, read, and approved the final manuscript.

## Conflict of Interest

The authors declare that the research was conducted in the absence of any commercial or financial relationships that could be construed as a potential conflict of interest.

## Publisher's Note

All claims expressed in this article are solely those of the authors and do not necessarily represent those of their affiliated organizations, or those of the publisher, the editors and the reviewers. Any product that may be evaluated in this article, or claim that may be made by its manufacturer, is not guaranteed or endorsed by the publisher.

## Footnotes

^1^ https://www.nsd.no/en

^2^ https://helseforskning.etikkom.no/reglerogrutiner/soknadsplikt/sokerikkerek?p_dim=34999&_ikbLanguageCode=us
