# Supplementary material for: Assessing Student Perceptions of a Norwegian University's COVID-19 Response Strategy: A Cross-Sectional Study
Source: Front Public Health. 2021 Aug 20;9:700542. doi: 10.3389/fpubh.2021.700542 (PMC8417720; doi:10.3389/fpubh.2021.700542)
Supplement: Supplementary file 1 [file Table_1.DOCX]

**Supplementary file 1.**

The factor constructs included in the survey and inspected through confirmatory factor analysis

| Factor label | Item label | Item statement | Item measurement |
| --- | --- | --- | --- |
| Risk Severity | Q3_1 | Covid-19 poses a serious threat to human health | 6-point Likert scale^a^ |
|  | Q3_2 | Covid-19 poses a serious threat to my own health | 6-point Likert scale^a^ |
|  | Q3_4 | Getting sick from Covid-19 is very serious | 6-point Likert scale^a^ |
|  | Q3_5 | Covid-19 causes severe damage to health | 6-point Likert scale^a^ |
| Risk Susceptibility | Q3_3 | It is likely that I will be exposed to Covid-19 | 6-point Likert scale^a^ |
|  | Q3_6 | I will probably get infected with Covid-19 | 6-point Likert scale^a^ |
| Attitudes IPC behaviours | Q4_1 | Keeping 1m distance is... | 6-point bipolar scale^b^ |
|  | Q5_1 | Keeping 1m distance is... | 6-point bipolar scale^c^ |
|  | Q4_2 | Washing hands often is… | 6-point bipolar scale^b^ |
|  | Q5_2 | Washing hands often is… | 6-point bipolar scale^c^ |
|  | Q4_3 | Using antibac is… | 6-point bipolar scale^b^ |
|  | Q5_3 | Using antibac is… | 6-point bipolar scale^c^ |
|  | Q4_4 | Staying at home if you are sick is… | 6-point bipolar scale^b^ |
|  | Q5_4 | Staying at home if you are sick is… | 6-point bipolar scale^c^ |
|  | Q4_5 | Cleaning equipment and workstations is… | 6-point bipolar scale^b^ |
|  | Q5_5 | Cleaning equipment and workstations is… | 6-point bipolar scale^c^ |
|  | Q4_6 | Sneezing and coughing into your elbow is… | 6-point bipolar scale^b^ |
|  | Q5_6 | Sneezing and coughing into your elbow is… | 6-point bipolar scale^c^ |
| Institutional trust | Q7_1 | I have trust in NMBU’s strategy to protect the students from Covid-19 exposure | 6-point Likert scale^a^ |
|  | Q7_2 | I believe that NMBU is applying the recommended outbreak response strategy by the Norwegian public health authorities | 6-point Likert scale^a^ |
|  | Q7_3 | The information we receive from NMBU concerning Covid-19 is trustworthy | 6-point Likert scale^a^ |
|  | Q7_4 | I believe that NMBU is doing everything they can to inform their students in a timely manner about Covid-19 | 6-point Likert scale^a^ |
| Attitudes reminders | Q8_1 | Stickers placed on the floor to encourage 1 meter of physical distancing are… | 6-point bipolar scale^b^ |
|  | Q9_1 | Stickers placed on the floor to encourage 1 meter of physical distancing are… | 6-point bipolar scale^c^ |
|  | Q8_2 | Posters placed near the sink that include information on how to properly wash hands are… | 6-point bipolar scale^b^ |
|  | Q9_2 | Posters placed near the sink that include information on how to properly wash hands are… | 6-point bipolar scale^c^ |
|  | Q8_6 | The visual display of campus-related infection control measures as a screen saver between lectures is… | 6-point bipolar scale^b^ |
|  | Q9_6 | The visual display of campus-related infection control measures as a screen saver between lectures is… | 6-point bipolar scale^c^ |
| Attitudes opportunities | Q8_3 | The visual display of antibac near the entrance of a building is… | 6-point bipolar scale^b^ |
|  | Q9_3 | The visual display of antibac near the entrance of a building is… | 6-point bipolar scale^c^ |
|  | Q8_5 | The visual display of antibac napkins in classroom is… | 6-point bipolar scale^b^ |
|  | Q9_5 | The visual display of antibac napkins in classroom is… | 6-point bipolar scale^c^ |
| Attitudes emails | Q8_4 | Email reminders sent by NMBU containing campus-related infection control measures are… | 6-point bipolar scale^b^ |
|  | Q9_4 | Email reminders sent by NMBU containing campus-related infection control measures are… | 6-point bipolar scale^c^ |
| Perceived behavioural control | Q10_1 | The placement of a sticker on the floor makes it easy for me to keep 1-meter distance when I enter a building | 6-point Likert scale^a^ |
|  | Q10_2 | Maintaining 1-meter distance remains my decision despite the placement of stickers on the floor | 6-point Likert scale^a^ |
|  | Q10_3 | The placement of posters by the sink makes it easy for me to wash my hands properly | 6-point Likert scale^a^ |
|  | Q10_4 | Washing my hands remains my decision despite the placement of posters by the sink | 6-point Likert scale^a^ |
|  | Q10_5 | The visual display of antibac by the entrance makes it easy for me to use when I enter a building | 6-point Likert scale^a^ |
|  | Q10_6 | Using antibac remains my decision despite the visual display by the entrance of a building | 6-point Likert scale^a^ |
|  | Q10_7 | Email reminders from NMBU containing campus-related infection control measures makes it easy for me to stay at home when sick | 6-point Likert scale^a^ |
|  | Q10_8 | Staying at home when sick remains my decision despite NMBU’s email reminders on campus-related infection control measures | 6-point Likert scale^a^ |
|  | Q10_9 | The visual display of antibac napkins in classroom makes it easy for me to clean the used equipment and workstation | 6-point Likert scale^a^ |
|  | Q10_10 | Cleaning the used equipment and workstation remains my decision despite the visual display of antibac napkins in the classroom | 6-point Likert scale^a^ |
|  | Q10_11 | The visual display of campus-related infection control measures as a screen saver between lectures makes it easy for me to remember to sneeze or cough into my elbow | 6-point Likert scale^a^ |
|  | Q10_12 | Sneezing or coughing into my elbow remains my decision, despite the visual display of campus-related infection control measures as a screen saver between lectures | 6-point Likert scale^a^ |

^a^ 1= strongly disagree; 2 = disagree; 3 = slightly disagree; 4 = slightly agree; 5 = agree; 6 = strongly agree

^b^ 1 = Unhelpful – 6 = Helpful

^c^ 1 = Unnecessary – 6 = Necessary
