# Supplementary material for: Assessing Student Perceptions of a Norwegian University's COVID-19 Response Strategy: A Cross-Sectional Study
Source: Front Public Health. 2021 Aug 20;9:700542. doi: 10.3389/fpubh.2021.700542 (PMC8417720; doi:10.3389/fpubh.2021.700542)
Supplement: Supplementary file 3 [file Table_3.DOCX]

**Supplementary file 3**

Test of homogeneity of variance through Levene’s test analysis

| Factor label | Item label | BF Statistic  *F* | df1 | df2 | Sig.  *p* |
| --- | --- | --- | --- | --- | --- |
| Risk Severity | Q3_1 | .987 | 1 | 338 | .321 |
|  | Q3_2 | 1.473 | 1 | 339 | .226 |
|  | Q3_4 | .317 | 1 | 338 | .574 |
|  | Q3_5 | 10.895 | 1 | 338 | .001 |
| Risk Susceptibility | Q3_3 | .124 | 1 | 338 | .725 |
|  | Q3_6 | 1.620 | 1 | 337 | .204 |
| Attitudes IPC behaviours | Q4_1 | .559 | 1 | 339 | .455 |
|  | Q5_1 | .502 | 1 | 339 | .479 |
|  | Q4_2 | .008 | 1 | 340 | .931 |
|  | Q5_2 | .101 | 1 | 337 | .750 |
|  | Q4_3 | .732 | 1 | 339 | .393 |
|  | Q5_3 | .543 | 1 | 338 | .462 |
|  | Q4_4 | .187 | 1 | 340 | .666 |
|  | Q5_4 | .288 | 1 | 339 | .592 |
|  | Q4_5 | .113 | 1 | 339 | .737 |
|  | Q5_5 | .225 | 1 | 339 | .635 |
|  | Q4_6 | .100 | 1 | 340 | .753 |
|  | Q5_6 | .728 | 1 | 337 | .394 |
| Institutional trust | Q7_1 | 2.219 | 1 | 337 | .137 |
|  | Q7_2 | .615 | 1 | 339 | .434 |
|  | Q7_3 | .151 | 1 | 340 | .698 |
|  | Q7_4 | .561 | 1 | 340 | .454 |
| Attitudes reminders | Q8_1 | .045 | 1 | 340 | .832 |
|  | Q9_1 | .828 | 1 | 333 | .364 |
|  | Q8_2 | .012 | 1 | 340 | .914 |
|  | Q9_2 | .481 | 1 | 333 | .489 |
|  | Q8_6 | .108 | 1 | 337 | .743 |
|  | Q9_6 | .010 | 1 | 333 | .919 |
| Attitudes opportunities | Q8_3 | .494 | 1 | 338 | .483 |
|  | Q9_3 | .148 | 1 | 334 | .701 |
|  | Q8_5 | .017 | 1 | 340 | .895 |
|  | Q9_5 | .002 | 1 | 334 | .962 |
| Attitudes emails | Q8_4 | .209 | 1 | 340 | .648 |
|  | Q9_4 | .392 | 1 | 334 | .531 |
| Perceived behavioural control | Q10_1 | .621 | 1 | 333 | .431 |
|  | Q10_2 | .026 | 1 | 335 | .872 |
|  | Q10_3 | 3.158 | 1 | 336 | .076 |
|  | Q10_4 | .304 | 1 | 333 | .581 |
|  | Q10_5 | .388 | 1 | 335 | .534 |
|  | Q10_6 | .819 | 1 | 336 | .366 |
|  | Q10_7 | 1.408 | 1 | 338 | .236 |
|  | Q10_8 | 1.295 | 1 | 337 | .256 |
|  | Q10_9 | .471 | 1 | 336 | .493 |
|  | Q10_10 | .014 | 1 | 336 | .905 |
|  | Q10_11 | .014 | 1 | 335 | .906 |
|  | Q10_12 | .332 | 1 | 333 | .565 |
